# How Eliminating Malaria May Also Prevent Iron Deficiency in African Children

**DOI:** 10.3390/ph11040096

**Published:** 2018-09-26

**Authors:** John Muthii Muriuki, Sarah H. Atkinson

**Affiliations:** 1 KEMRI-Wellcome Trust Research Programme, 80108 Kilifi, Kenya; 2 Centre for Tropical Medicine and Global Health, Nuffield Department of Medicine, University of Oxford, Oxford OX3 7FZ, UK; 3 Department of Paediatrics, University of Oxford, Oxford OX3 9DU, UK

**Keywords:** malaria, iron deficiency, hepcidin, TNF, children, Africa

## Abstract

Malaria and iron deficiency are common among children living in sub-Saharan Africa. Several studies have linked a child’s iron status to their future risk of malaria infection; however, few have examined whether malaria might be a cause of iron deficiency. Approximately a quarter of African children at any one time are infected by malaria and malaria increases hepcidin and tumor necrosis factor-α concentrations leading to poor iron absorption and recycling. In support of a hypothetical link between malaria and iron deficiency, studies indicate that the prevalence of iron deficiency in children increases over a malaria season and decreases when malaria transmission is interrupted. The link between malaria and iron deficiency can be tested through the use of observational studies, randomized controlled trials and genetic epidemiology studies, each of which has its own strengths and limitations. Confirming the existence of a causal link between malaria infection and iron deficiency would readjust priorities for programs to prevent and treat iron deficiency and would demonstrate a further benefit of malaria control.

## 1. Introduction

Malaria and iron deficiency are important public health problems especially in developing countries [1,2]. In 2016, malaria caused an estimated 216 million cases of sickness and 445,000 deaths (91% in sub-Saharan Africa) [2]. Among African children under the age of five years, malaria caused an estimated 292,000 deaths in 2015 [3]. The disease has remained persistent and widespread across sub-Saharan Africa (SSA), affecting 24% of the population at any one time [4]. Similarly, iron deficiency is common in SSA, where it affects more than half of children [5,6]. Iron deficiency is associated with poor child growth including impaired brain development and long-term impairment of behavioral and cognitive performance [7,8,9]. Furthermore, iron deficiency is the main cause of anemia and iron deficiency anemia (IDA) is the leading cause of years lived with disability in children [1].

Iron supplements are inexpensive and widely used for the prevention and treatment of iron deficiency in African children. However, there are long-standing concerns regarding the safety of iron supplementation [10,11]. A large trial in Pemba, Tanzania reported an increased risk of malaria-related events among the group supplemented with iron [12] and other trials have reported inconsistent findings [13,14,15,16]. In 2016, a Cochrane review reported that iron supplementation was not associated with an increased risk of clinical malaria when malaria prevention and management services were provided [17]. Thus, the World Health Organization (WHO) updated its recommendations for iron supplements and micronutrient powders in malaria endemic areas to include supplementation when the prevalence of anemia is 40% or higher in conjunction with malaria control and management practices [18]. However, questions remain regarding how adequate malaria control and prevention measures need to be before iron supplementation is deemed safe in resource-limited settings [3]. Moreover, prospective cohort studies have indicated that iron replete children may be at increased risk from malaria infection [19,20,21,22,23]. In addition to the risk of malaria, iron supplements/fortification may not be absorbed in children with malaria and hence would be ineffective [24] and have also been associated with pathogenic gut microbiota and bacterial infection [25]. New strategies are therefore needed to prevent and treat iron deficiency. In this paper we outline the hypothesis that malaria could contribute to the burden of iron deficiency in children living in SSA.

## 2. The Malaria Iron Deficiency Hypothesis

We hypothesize that malaria may be causally linked to iron deficiency in African children by increasing concentrations of the iron hormone hepcidin, as well as increasing inflammatory cytokines, such as tumor necrosis factor-α (TNF-α). In SSA, up to 50% of children may be asymptomatically infected with malaria and 24% have febrile malaria at any one time [4,26]. Asymptomatic individuals carry malaria parasites but are unlikely to seek medical attention and there may be delays in the treatment of febrile malaria and other illnesses in resource-limited settings. Many children are therefore likely to have chronically up-regulated hepcidin, and inflammatory cytokines such as TNF-α, which in turn block the absorption and recycling of iron. Indeed a study in Ivorian children showed that iron absorption was halved in children with afebrile malaria and increased when malaria infection was treated [24].

The iron hormone hepcidin may link malaria with iron deficiency. Both clinical and asymptomatic malaria infections increase hepcidin concentrations. Clinical episodes of *Plasmodium falciparum* malaria are associated with markedly increased hepcidin concentrations in African children [27,28,29,30,31]. Similarly, even asymptomatic *P. falciparum* is associated with a doubling of hepcidin concentrations [31,32]. Treatment of malaria significantly reduces hepcidin concentrations [28,32,33]. Furthermore, the up-regulatory effects of malaria on hepcidin concentrations appear to occur both in the presence and absence of inflammation suggesting that malaria may further increase hepcidin independently of inflammation [31]. The mechanisms through which malaria up-regulates hepcidin production are not fully elucidated, but may include the bone morphogenetic protein (BMP)/sons of mothers against decapentaplegic (SMAD) pathways [34].

**Figure 1:**
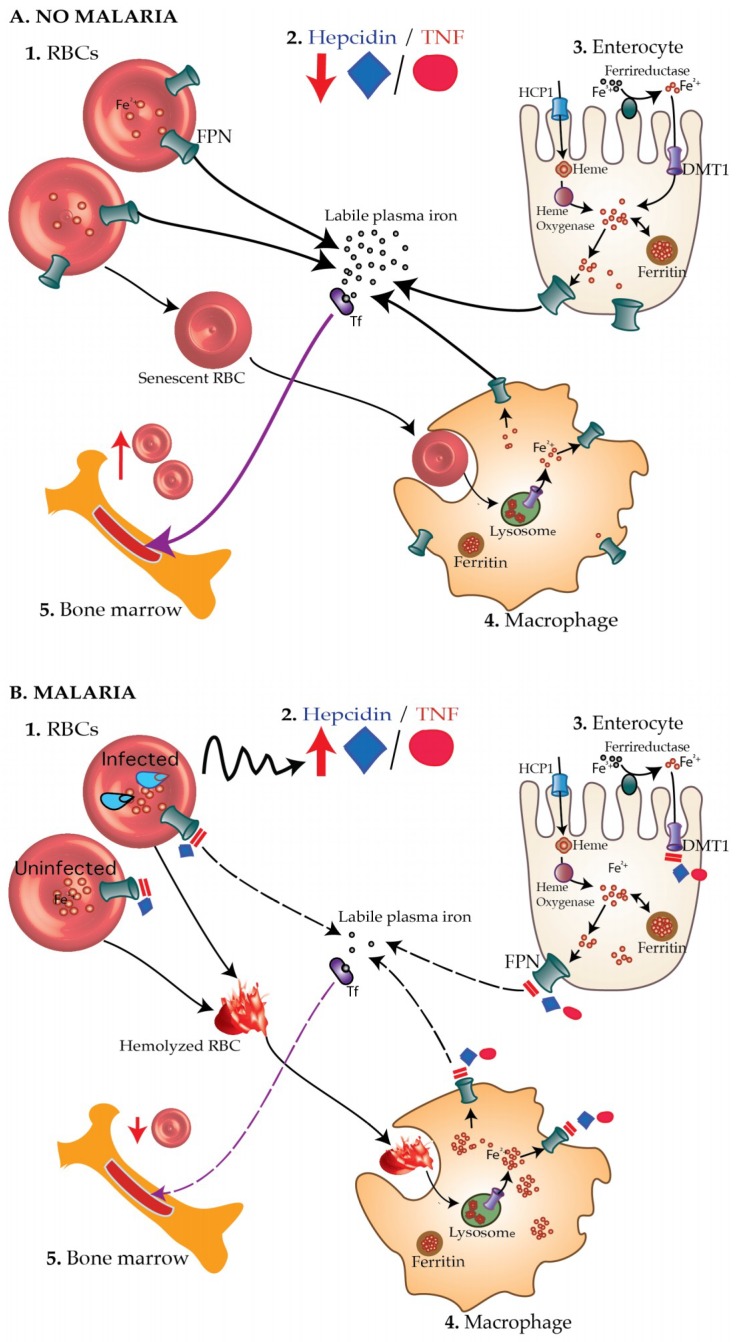
The malaria—iron deficiency hypothesis. (**A**) In healthy children without malaria (**A1**), concentrations of hepcidin and TNF-α are low (**A2**) leading to increased absorption of iron through enterocytes (**A3**), reduced hemolysis of RBCs and increased recycling of iron recovered from senescent RBCs by macrophages (**A4**). More iron is thus available for the production of new RBCs (**A5**). (**B**) On the other hand, during malaria infection, blood-stage malaria parasites (**B1**) elicit increased production of hepcidin and TNF-α (**B2**), which, in turn, block absorption of iron through DMT1 and ferroportin (FPN) on enterocytes (**B3**). Hepcidin also degrades ferroportin on both infected and uninfected RBCs leading to accumulation of intracellular iron, oxidative stress, and consequently hemolysis. Hemolyzed RBCs are taken up by the macrophage (**B4**). Hepcidin and TNF-α inhibit recycling of iron recovered from hemolyzed RBCs back into the circulation leading to deficiency of the amount of biologically available iron. Consequently, little iron is available to produce new RBCs by the bone marrow leading to iron deficiency anemia (**B5**). Tf, transferrin.

Increased hepcidin concentrations due to frequent and chronic malaria parasitemia in children with iron-poor diets may lead to iron deficiency. Hepcidin prevents iron absorption and recycling by inhibiting the activity of ferroportin [35], the sole known iron exporter which is abundant in macrophages [36,37], enterocytes and hepatocytes [36,38,39], as well as erythrocytes [40]. Hepcidin also degrades and inhibits the transcription of divalent metal transporter 1 (DMT1), thus blocking iron absorption through the duodenal enterocytes [41,42]. Furthermore, hepcidin blocks iron export from red blood cells (RBCs) leading to accumulation of iron, oxidative stress and hemolysis [40]. This may explain why both infected and uninfected RBCs burst during malaria infection leading to anemia (and probably further spread of the malaria parasites). Figure 1 illustrates how malaria-induced hepcidin might contribute to iron deficiency.

Malaria may also cause iron deficiency through increasing inflammatory cytokines such as tumor necrosis factor-α (TNF-α). Uncomplicated and asymptomatic malaria significantly raise TNF-α concentrations [43,44]. TNF-α blocks iron recycling from macrophages and inhibits erythropoiesis [45,46]. In addition, TNF-α, independently of hepcidin, blocks intestinal iron absorption by reducing DMT1 expression [47], increasing deposition of iron into ferritin and degrading ferroportin [48]. In a cohort of Gambian children, the TNF_−308_ AA genotype (which is associated with higher TNF-α transcription compared with TNF_−308_ AG and TNF_−308_ GG genotypes [49,50]) was strongly associated with increased risk of iron deficiency and IDA [51]. Interestingly, this effect was observed at the end of a malaria season when the prevalence of clinical malaria was highest [51]. Furthermore, zinc protoporphyrin concentrations were significantly raised in the TNF_−308_ AA genotype indicating dyserythropoiesis [51].

Several studies support the hypothesis that malaria causes iron deficiency. A study in Kenyan and Gambian children observed that the prevalence of iron deficiency and IDA was markedly higher at the end of a malaria season compared to the start [52]. Interruption of malaria transmission in the Kenyan highlands with antimalarials and indoor residual spraying reduced the prevalence of iron deficiency from 36% to 25% and more than halved the prevalence of IDA (from 27% to 12%) [53]. However, the observational nature of these studies does not necessarily imply a causal relationship. Additionally, a meta-analysis of intermittent preventive treatment (IPT) of malaria reported a 29% reduction of anemia in children following treatment [54]. Moreover, in Ivorian children, treatment of asymptomatic malaria significantly reduced hepcidin concentrations and inflammation and doubled iron absorption [24].

A key difficulty is distinguishing between uncomplicated IDA and the anemia of inflammation (AI) among children living in sub-Saharan Africa where malaria and other infections are highly prevalent. This challenge can be addressed by measuring a wide range of iron markers [55] including hepcidin [56]. Hepcidin was demonstrated to be significantly lower in children with IDA compared to those with AI [56]. Other markers of iron status that can be used to discriminate IDA and AI include soluble transferrin receptors (sTfR) and total iron binding capacity (TIBC), which are elevated during IDA compared to AI, or serum ferritin, which is normally decreased during IDA and increased during AI [55]. However, the effects of inflammation or infection on iron status may obscure the true prevalence of IDA in African children [57].

## 3. Testing the Hypothesis

How can the hypothesis be tested? Below, we look at potential study designs as well as their strengths and limitations.

### 3.1. Observational Studies

Longitudinal cohort studies following up malaria exposed and unexposed children for iron status would allow investigation of whether malaria causes iron deficiency. However, since individuals in malaria endemic areas are likely to be exposed to malaria repeatedly and in varying degrees over time [4], it would be difficult to group them as exposed or unexposed with certainty. Individuals who are continuously exposed to malaria develop partial immunity to malaria and may be misclassified as unexposed [58]. Moreover, it is difficult to specifically determine the degree of malaria exposure [59,60]. Another challenge is the fact that host iron status may also influence malaria risk making reverse causality a possibility [19,20,21]. Pragmatic cohort studies would involve comparing iron deficiency during the course of malaria seasons although many other factors may influence iron status during that period [52]. For example, the nutritional status of children may improve during harvest seasons, which may coincide with rainy seasons (or peak malaria transmission) thereby confounding a possible effect of malaria on iron status.

Another approach would be to spatially and temporally map-out the distribution of malaria and iron deficiency. If malaria causes iron deficiency, then areas or periods of high malaria transmission would also be associated with higher prevalence of iron deficiency. Carefully gathered and mapped epidemiological data of malaria in Africa, both in space and time, are available [4,61]. Likewise, a number of iron deficiency studies have been conducted in African children over the years although not mapped. However, this approach is limited by the fact that some markers of iron especially ferritin and soluble transferrin receptors (which are commonly measured), are raised during malaria infection [62,63,64]. Thus, malaria may obscure the true picture of iron deficiency so that children in malaria regions may appear more iron replete. Social economic status may further confound the geographical distribution of malaria and iron deficiency since both are more likely to occur in the poorest communities.

### 3.2. Randomized Controlled Trials

Randomized controlled trials (RCTs) remain the gold standard study designs for investigating a causal relationship. Individuals could be randomized to receive interventions known to be effective against malaria such as antimalarials and insecticide-treated bed nets and then iron status could be assessed after a period of time. For example, children could be randomized to receive intermittent preventive treatment (IPT) of malaria followed by assessment of iron status. A few previous trials have reported a non-significant improvement in concentrations of ferritin [13,65] and a decrease in sTfR [14] following IPT. However, both ferritin and sTfR are raised during malaria infection [62,63,64] making interpretation of iron status difficult and thus they may not be the best indicators of the effect of IPT on iron status. Transferrin saturation may be a good indicator of improved iron absorption while reduced ZPP concentrations may indicate improved erythrocyte iron incorporation. However, it may be difficult to justify large trials randomizing children to either malaria prevention/treatment or none.

### 3.3. Mendelian Randomization Studies

Another approach is Mendelian randomization (MR) which utilizes genetic variants as proxies for modifiable environmental exposures (or instrumental variables) to infer a causal relationship between an exposure and an outcome [66]. This study design provides an alternative to RCTs since genetic variants are unlikely to be confounded by environmental factors and reverse causality is eliminated as genetic variants are allocated at conception [67]. MR also reflects a life-time of exposure, which is important since age is a critical determinant of infectious risk. This approach has been successfully employed in other disease processes and has helped to explain previous controversies [68,69,70].

**Figure 2:**
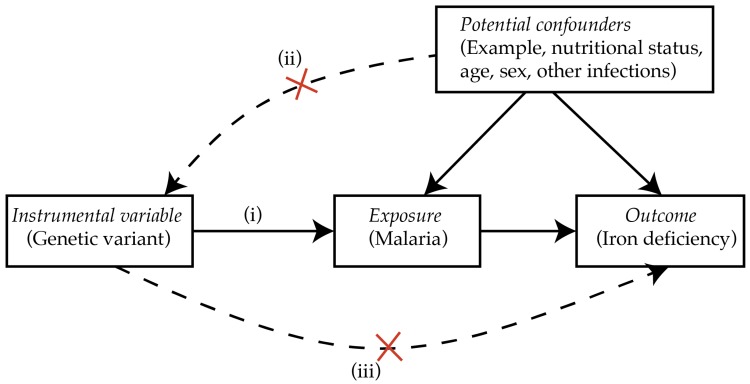
Conceptual MR causal inference framework. (i) Genetic variants reliably associated with malaria are required, for example, the sickle cell trait. (ii) The genetic variant should not be associated with any measured potential confounders. (iii) The genetic variant should influence iron deficiency only in populations at risk of malaria. Adapted from Sheehan et al. [75].

Similarly, MR can be utilized to study whether malaria causes iron deficiency. There are known genetic polymorphisms that are associated with resistance to malaria. For example, sickle cell trait which results from inheritance of one abnormal allele of the beta-globin gene is associated with 50% protection against uncomplicated clinical malaria and 86% protection against severe malaria [71]. Alpha- and beta-thalassemias, glucose-6-phosphodehydrogenase (G6PD), ABO blood group, and glycophorins are additional genes known to be associated with resistance to severe malaria [72]. If malaria causes iron deficiency, then polymorphisms that protect from malaria would also be associated with reduced risk of iron deficiency. However, the polymorphisms should only influence iron status through malaria, thus the effect of the polymorphism(s) on iron status should only be observed in populations at risk of malaria but not in malaria-free populations (i.e., there should be no pleiotropy or independent effect of the polymorphisms on iron status). Additionally, each of the protective polymorphisms should influence iron deficiency in the same direction [73,74]. Figure 2 illustrates the conceptual MR causal diagram for malaria and iron deficiency.

## 4. Implications of the Hypothesis

Since individuals in malaria-endemic regions are likely to have chronically up-regulated hepcidin concentrations, the current efforts of iron supplementation in these populations may not address the problem of iron deficiency. Raised hepcidin levels in malaria-infected individuals would not only block iron absorption [24] but also utilization [76,77,78]. A systematic review of randomized controlled trials evaluating the effect of iron supplementation on hemoglobin/anemia in children reported limited gains in malarial hyperendemic areas [76]. Furthermore, unabsorbed iron due to malaria infection may disturb gut microbiota leading to gastrointestinal disorders [79,80,81]. In the largest iron supplementation trial in Pemba, Tanzania, increased risk of adverse events were reported among children in the supplemented arm [12]. Thus, the effectiveness and safety of iron supplementation or fortification in malaria-endemic regions has remained questionable.

If, indeed, malaria causes iron deficiency, then strategies aimed at malaria elimination may also address iron deficiency. Causality in the malaria-iron deficiency relationship could be tested using randomized trials of interventions that protect against malaria, such as IPT trials, or by Mendelian randomization studies where randomization would be by genetic variants that protect from malaria, such as the sickle cell trait. Advantages of Mendelian randomization are that genes confer life-long protection against malaria and that it may be unethical to randomize children to not receive an intervention of proven efficacy. Furthermore, our hypothetical concept could also be extended to other causes of chronic infection, which may contribute to the burden of iron deficiency through inflammation-induced up-regulation of hepcidin. Confirmation of a role of malaria and other infections in causing iron deficiency could lead to a readjustment of priorities for public health programs to prevent and treat iron deficiency in sub-Saharan Africa. Thus, we recommend further studies to test the malaria-iron deficiency hypothesis and suggest that control of malaria and other infections could be utilized as an additional strategy to improve the iron status of children living in Africa.
